# Biodegradation of polyhydroxyalkanoates: current state and future prospects

**DOI:** 10.3389/fmicb.2025.1542468

**Published:** 2025-02-24

**Authors:** Ani Paloyan, Mane Tadevosyan, Diana Ghevondyan, Lev Khoyetsyan, Mariam Karapetyan, Armine Margaryan, Garabed Antranikian, Hovik Panosyan

**Affiliations:** ^1^Scientific and Production Center “Armbiotechnology” National Academy of Science of Armenia, Yerevan, Armenia; ^2^Research Institute of Biology, Biology Faculty, Yerevan State University, Yerevan, Armenia; ^3^Department of Biochemistry, Microbiology and Biotechnology, Yerevan State University, Yerevan, Armenia; ^4^Center of Biobased Solutions (CBBS), Institute of Technical Biocatalysis, Hamburg University of Technology, Hamburg, Germany

**Keywords:** polyhydroxyalkanoates (PHAs), biodegradation, PHA depolymerases, extremophiles, circular bioeconomy

## Abstract

Polyhydroxyalkanoates (PHAs) are biobased and biodegradable polymers that offer a sustainable alternative to conventional plastics, addressing the escalating concerns over plastic pollution. While their environmental advantages are well-documented, the efficient degradation of PHAs in natural and engineered environments remains a critical component of their lifecycle. This review provides a comprehensive overview of PHA-degrading bacteria isolated from diverse ecosystems and highlights the pivotal role of PHA depolymerases in achieving PHA circularity. Microbial adaptation to diverse environmental conditions, such as extreme temperatures, salinity, and pH, significantly influences enzymes properties, including the stability, activity, and substrate specificity of PHA-degrading enzymes. These adaptations often enhance enzyme, performance, enabling functionality under challenging conditions. Consequently, extremophilic microorganisms are invaluable resources for discovering and engineering robust PHA depolymerases for industrial and environmental applications. This review underscores the urgent need for further research to improve the ecological and economic sustainability of PHA waste management.

## Introduction

The growing global reliance on plastics has created significant environmental challenges, primarily due to their persistence in natural ecosystems and the difficulties associated with their disposal (Covello et al., 2024). Conventional petrochemical-based plastics degrade extremely slowly, accumulating in terrestrial and aquatic environments and contributing to widespread pollution. To address these issues, bioplastics have emerged as sustainable alternatives, offering the potential to reduce environmental harm while retaining the versatility of traditional plastics (Yadav and Nikalje, 2024).

Among the various types of bioplastics, polyhydroxyalkanoates (PHAs) have garnered significant attention as promising biodegradable polymers. Synthesized by a wide range of microorganisms as intracellular carbon and energy reserves, PHAs are renewable, biodegradable, and environmentally friendly. These properties make PHAs particularly appealing for combating global plastic pollution (Getino et al., 2024). Furthermore, PHAs are biocompatible, enabling their application in medical fields such as drug delivery systems and surgical implants, thus broadening their potential uses.

However, the successful adoption of PHAs depends on a thorough understanding of their complete lifecycle, particularly their degradation in natural and engineered environments. PHA breakdown is essential for reintegrating these materials into natural biogeochemical cycles. This process heavily relies on microbial activity, where specialized bacteria and fungi produce extracellular depolymerases to hydrolyze PHA polymers into monomers and oligomers, which can then serve as carbon sources for microbial growth (Ray and Kalia, 2017). These microorganisms are found in diverse environments, including terrestrial soils, freshwater, marine ecosystems, and extreme conditions like geothermal springs and cryogenic soils, demonstrating their ecological adaptability.

The enzymatic degradation of PHAs is a critical factor in their biodegradability. Enzymes such as PHA depolymerases play a pivotal role in this process by hydrolyzing the ester bonds within PHA polymer chains, generating products that microorganisms can further metabolize (Millan and Hanik, 2023). PHA depolymerases exhibit remarkable specificity and efficiency, targeting short-chain-length (SCL-PHA) or medium-chain-length (MCL-PHA) polymers (Knoll et al., 2009). The efficiency of these enzymes is influenced by factors such as polymer composition, crystallinity, environmental conditions, and the presence of cofactors (Bhatt et al., 2011).

Despite significant progress in understanding PHA-degrading microorganisms and their enzymes, several knowledge gaps persist. For example, further research is needed to elucidate the interactions between environmental factors (e.g., pH, temperature, salinity) and enzymatic activity. Additionally, investigating the mechanisms underlying microbial adaptation to extreme environments, such as hot springs or cryogenic soils, could uncover novel biocatalysts. Addressing these gaps will enhance the development of efficient waste management systems and industrial processes for bioplastic recycling.

This review provides a comprehensive overview of the diversity and ecological distribution of PHA-degrading bacteria and their enzymes. It highlights the enzymatic mechanisms involved in PHA degradation, the structural and functional characteristics of depolymerases, and the factors influencing their activity. Furthermore, it explores the potential applications of PHA-degrading microorganisms and their enzymes in bioplastic recycling, waste management, and sustainable biotechnology. By synthesizing recent advances, this review aims to guide future research and innovation in leveraging microbial systems to address the global plastic crisis.

## Degradation of polyhydroxyalkanoates

PHAs are biodegradable polymers that undergo degradation through various mechanisms, including photooxidative, catalytic, thermal, and mechanical degradation. Among these, microbial degradation plays a crucial role in the complete breakdown of PHAs in natural environments.

Photooxidative degradations occurs when PHA is exposed to light, particularly UV radiation. The energy from light causes the formation of free radicals within the polymer chains, leading to chain scission and degradation. This process is relatively slow and highly dependent on the intensity and duration of UV exposure (Lalonde et al., 2025).

Catalytic degradation involves the use of catalytic entities, including enzymes, transition metal ions (for the Fenton reaction), nanozymes are used to accelerate the breakdown of PHA. Catalysts specifically target the ester bonds in PHA, leading to faster degradation. However, the requirement for specific catalysts can make the process more complex and costly (Zandieh et al., 2024).

Thermal degradation occurs at high temperatures causing random chain scission in PHA polymer chains. This process results in the formation of smaller molecules, such as oligomers and monomers. While thermal degradation can be effective, it often produces unwanted byproducts and requires substantial energy input (Kunioka and Doi, 1990; Hablot et al., 2008).

Mechanical degradation involves physical forces such as grinding, shear stress, or mechanical wear that break down PHA polymers. This process primarily leads to fragmentation rather than chemical degradation. However it does not result in complete molecular breakdown but rather physical disintegration (Lin et al., 2020).

Microbial degradation of PHA is widely distributed among bacteria. PHA-degrading bacteria have been identified in diverse environmental niches, including soil (geothermal and cryogenic), hot springs, freshwater, and marine ecosystems, indicating the widespread distribution of PHA-degrading microorganisms (Hori et al., 2020; Volova et al., 2010; Boyandin et al., 2012; Takeda et al., 2002). These microorganisms play a vital role in the natural biodegradation of PHAs.

### PHA degrading bacteria isolated from soil and sludge

Soil environments harbor a diverse array of PHA-degrading bacteria across various genera, including *Acidovorax*, *Acinetobacter*, *Arthrobacter*, *Bacillus*, *Burkholderia*, *Cytophaga*, *Rhodococcus, Cupriavidus*, *Mycobacterium, Nocardiopsis*, *Pseudomonas, Stenotrophomonas, Paraburkholderia*, *Streptomyces, Rhizobium*, *Variovorax*, *Xanthomonas*, *Alcaligenes, Lihuaxuella, Thermus, Schlegelella, Paenibacillus* and *Zoogloea* (Hori et al., 2020; Boyandin et al., 2012; Volova et al., 2006; Prudnikova et al., 2021; Thomas et al., 2022; Pham et al., 2024). These bacteria exhibit significant functional diversity, with some strains demonstrating rapid degradation rates under laboratory conditions. For instance, *Pseudomonas* sp. DSDY0501, isolated from activated sludge, completely degraded poly (3-hydroxybutyrate) (PHB) films within 21 h in liquid culture, utilizing PHB as its sole carbon source (Di et al., 2019). Similarly, strains such as *Bacillus subtilis* PLA 2.3.1 and PET 2.2.1, *Streptomyces griseorubens* ACTY2-2, *Rhizobium pusense* PLA1-1, *Priestia aryabhattai* (A13, A34, L5, N7), isolated from plastic-contaminated soils in Armenia, have demonstrated the ability to degrade PHBV and PHBH. Additionally, *Priestia aryabhattai* A34, *Priestia megaterium* L7 and N7 were found to be capable of degrading PHB polymers (Khoyetsyan et al., 2024; Tadevosyan et al., 2024).

PHAs, biodegradable bioplastics, produced by various microorganisms as intracellular carbon and energy reserves (Arnosti, 2011), have also been targeted by *Pseudomonas* strains isolated from soil and compost. These strains exhibit promising potential for bioremediation and waste management, particularly in composting systems where they facilitate PHA degradation (Jaeger and Rosenau, 2004; Spasic et al., 2018).

The ability of *Pseudomonas* species to degrade PHAs, coupled with their production of specific depolymerases, highlights their importance in the environmental breakdown of bioplastics and the recycling of biodegradable waste (Arnosti, 2011; Jaeger and Rosenau, 2004).

Interestingly, the homopolymer poly-3-hydroxybutyrate (poly-3-HB) degraded faster than its copolymer counterpart, poly-3-HB/3-HV, demonstrating the impact of polymer composition on biodegradability. Additionally, microbial communities associated with the polymers are habitat-specific and differ significantly from those in the surrounding soil. Key bacterial genera such as *Burkholderia*, *Bacillus*, and *Cupriavidus*, alongside fungal species like *G. butleri* and *Penicillium* sp., have been identified as active degraders.

The interplay between environmental conditions and microbial ecosystems significantly influences PHA degradation, contributing to the variability of biodegradation rates across different geographies (Boyandin et al., 2013).

### PHA degrading bacteria isolated from aquatic environments

While soil environments host a diverse array of PHA-degrading bacteria with significant functional diversity, marine ecosystems also provide a unique habitat for these microorganisms, requiring adaptations to salinity and host interactions. Genera such as *Enterobacter*, *Bacillus*, *Comamonas* and *Gracilibacillus* have been identified as key PHA degraders in seawater and marine habitats (Volova et al., 2010; Boyandin et al., 2012; Volova et al., 2006; Schirmer et al., 1993).

Additional PHA degraders include *Alcaligenes faecalis* (from activated sludge), *Ilyobacter delafieldii* (from lake water and estuarine sediment), *Caldimonas manganoxidans* (from hot springs), and other species isolated from anaerobic sewage sludge (Takeda et al., 2002; Schirmer et al., 1993). Interestingly, some strains have been isolated from marine organisms, such as *Acidovorax* spp. from Siberian sturgeon, *Acinetobacter* spp. from European sea bass, and *Ochrobactrum* spp. from giant river prawns (Liu et al., 2010).

Studies have also identified microbial degradation of poly (3-hydroxybutyrate-co-3-hydroxyhexanoate) (PHBH) polymers by strains such as *Glaciecola lipolytica* E11, *Aestuariibacter halophilus* S23, and *Pseudoalteromonas lipolytica* S35. These microbes form biofilms on PHBH polymer surfaces within 3 days of incubation at 23°C in seawater. Notably, these strains do not degrade other types of bioplastics, demonstrating specificity toward PHBH. The formation of clear zones observed around colonies on PHBH-containing agar plates further confirm their PHA-degrading activity (Morohoshi et al., 2018).

Several bacterial species isolated from various marine environments have been identified as capable of degrading PHA, with *Pseudomonas* species playing a particularly prominent role in this process. *Pseudomonas aeruginosa*, *Pseudomonas fluorescens*, and *Pseudomonas putida* have been shown to produce extracellular PHA depolymerases —enzymes that break down PHA into smaller monomers, which can then be utilized by bacteria as a carbon source (Arnosti, 2011).

A novel tropical marine bacterium, *Pseudomonas* sp. NRRL B-300083, has demonstrated the ability to degrade PHB/PHV copolymers in seawater. Similarly, denitrifying bacteria such as *Comamonas testosteroni* and certain strains of the *Variovorax* genus have been found to degrade PHB and its copolymers, with faster degradation observed in copolymers containing higher proportions of hydroxyvalerate (Volova et al., 2006).

### PHA degrading bacteria isolated from extremophilic environments

Extremophilic habitats, such as geothermal springs and cryogenic soils, host bacteria that exhibit remarkable adaptations to challenging conditions, including extreme temperatures, acidic or basic pH, and high salinity. These environments exert unique selection pressures that drive the development of specialized microbial adaptations. For example, *Microbulbifer* sp. SOL03 and *Bacillus vietnamensis* SOL04 demonstrate PHA-degrading activity across a wide temperature range (10–42°C), while *Halobacillus trueperi SOL01* tolerates salinity levels as high as 15% NaCl (Park et al., 2021). Additionally, halophilic actinomycetes such as *Nocardiopsis aegyptia* efficiently degrade PHB and PHBV copolymers, achieving up to 89.94% weight loss within 30 days (Ghanem et al., 2005).

Thermophilic bacteria capable of degrading PHBH polymers have also been isolated from geothermal springs. For instance, *Anoxybacillus* sp. K99 and *Anoxybacillus karvacharensis* K1, isolated from the Karvachar geothermal spring in Nagorno-Karabakh*, Parageobacillus toebii* H-70 isolated from Hankavan hot spring, have demonstrated the ability to degrade PHBH at 55°C (Khoyetsyan et al., 2024; Tadevosyan et al., 2024).

Cryogenic soils in Siberia’s subarctic regions have yielded PHB-degrading bacteria such as *Bacillus pumilus, Paraburkholderia* sp.*, Pseudomonas* sp.*, Rhodococcus* sp.*, Stenotrophomonas rhizophila, Streptomyces prunicolor,* and *Variovorax paradoxus* (Prudnikova et al., 2021). Among these, *Ralstonia* sp. exhibited the highest specific activity, maintaining functionality even at low pH levels (3.3–3.7).

The ability of PHA-degrading bacteria to thrive in extreme conditions is closely tied to the specialized characteristics of their enzymes, such as enhanced temperature and pH stability. These unique adaptations, evident across diverse extremophilic habitats, highlight the untapped potential of these strains for industrial applications under harsh conditions. By bridging the gap between microbial ecology and biotechnology, extremophilic PHA-degrading bacteria offer innovative solutions for industrial processes and waste management in challenging environments.

## Analytical methods for assessing microbial degradation of plastics

Several analytical methods are employed to study the microbial degradation of plastics, particularly bioplastics like PHAs. These methods can be categorized based on their purpose, such as visual assessment, structural analysis, chemical analysis, and molecular approaches.

Microbial degradation of bioplastics is often evidenced by the formation of clear zones around microbial colonies on plates where bioplastics serve as the sole carbon source. The diameter of this zone is commonly used to quantitative measure of biodegradation efficiency (Tezuka et al., 2004; Lee et al., 2005; Kumaravel et al., 2010). Advanced analytical techniques, such as scanning electron microscopy (SEM) and Fourier Transform Infrared (FTIR) spectroscopy, play crucial roles in understanding the structural and molecular changes occurring during microbial degradation. SEM is widely used to visualize the breakdown of PHAs, revealing surface irregularities and structural instability caused by microbial activity (Wu, 2009; Tachibana et al., 2013). FTIR spectroscopy complements SEM by detecting changes in bond intensities, providing insights into the enzymatic action on polymer chains.

A summary of the methods used for assessing plastic degradation is presented in Table 1.

**Table 1:** Comprehensive list of commonly used methods to study the microbial degradation of bioplastics.

Method	Category	Purpose	Quantitative/Qualitative	Reference
Clear zone assay	Visual assessment	Detect PHA degradation	Qualitative (zone diameter)	Jendrossek and Handrick (2002)
SEM, TEM, AFM	Structural analysis	Visualize surface changes	Qualitative & Quantitative	Tachibana et al. (2013), Tokiwa and Calabia (2004)
FTIR, NMR	Chemical analysis	Detect bond changes	Qualitative & Quantitative	Valle Iulianelli et al. (2016), Bonartsev et al. (2007)
GC–MS, HPLC	Chemical analysis	Identify degradation products	Quantitative (monomers)	Rosato et al. (2022), Sintim et al. (2020)
Differential scanning calorimetry, Thermogravimetric analysis	Thermal analysis	Measure thermal properties	Quantitative (Tm, Td)	Persico et al. (2012)
Enzyme assays	Enzyme activity	Detect depolymerase activity	Quantitative (U/mL)	Vigneswari et al. (2015)
Metagenomics	Molecular analysis	Identify genes	Quantitative (gene abundance)	Zhang et al. (2021), Viljakainen and Hug (2021)
qPCR	Molecular analysis	Detect depolymerase genes	Quantitative (gene copy)	Zampolli et al. (2024)

## Influence of additives and environmental factors on microbial PHA degradation

The efficiency of PHA biodegradation is influenced by several factors, including the polymer’s physicochemical properties and environmental conditions. Key polymer properties affecting degradation include shape, size, molecular weight, functional groups, crystallinity, hydrophobicity or hydrophilicity, and the presence of additives. Environmental factors such as temperature, light, oxygen availability, salinity, pH, and nitrate concentrations also play significant roles. Abiotic factors, such as temperature and light, enhance polymer accessibility to microbial degradation, while biotic factors, particularly enzyme activity, directly drive the breakdown process (Volova et al., 2010; Boyandin et al., 2013; Bonartseva et al., 2002). For instance, increased salinity typically reduces degradability, whereas higher temperatures and light levels promote it.

Seasonal studies conducted in the Sea of Japan and the Baltic Sea have demonstrated that PHA degradation rates are higher during the summer, driven by increased microbial activity under warmer temperatures (Zandieh et al., 2024; Tokiwa and Calabia, 2004). PHB degradation was most effective under aerobic conditions, with minimal degradation observed in anaerobic environments. The addition of nitrate further enhanced degradation across all aeration levels by stimulating denitrifying microbes. Notably, PHB degradation without nitrate occurred at 20°C but was inhibited at 5°C, whereas nitrate supplementation enabled degradation even at 5°C (Bonartseva et al., 2002).

Tropical soils also exhibit higher PHA degradation rates due to favorable climatic conditions, including elevated humidity and diverse microbial communities (Li et al., 2016).

Studies conducted in Lake Shira, a saline lake in Russia, revealed that PHA degradation occurs under both oxygen-rich (aerobic) and oxygen-deprived (anaerobic) conditions,. Under aerobic conditions, microorganisms convert PHAs into carbon dioxide and water, whereas anaerobic conditions yield methane and carbon dioxide (Volova et al., 2010).

The PHB degradation performance of *Cutibacterium* sp. SOL05 was notable, achieving 66% degradation within 7 days and 74% within 10 days. The addition of carbon sources significantly enhanced this efficiency. Among tested carbon sources-glucose, galactose, sucrose, xylose, lactose, and fructose-lactose had the most pronounced effect. A 1% lactose concentration increased degradation by 1.5 times compared to the control (Park et al., 2021).

The efficiency of PHA biodegradation is closely linked to the polymer’s physical and chemical properties. The degradation rate primarily depends on (i) crystallinity, (ii) the type of polymer (homopolymer vs. copolymer), and (iii) the copolymeric structure. Due to its high crystallinity, PHB is generally more resistant to biodegradation. Copolymers such as PHBV, which incorporate hydroxyvalerate into PHB, degrade more readily due to their increased amorphous regions. A higher hydroxyvalerate content reduces crystallinity, thereby enhancing the polymer’s biodegradability by facilitating enzyme adhesion and accelerating degradation (Meereboer et al., 2020). Films with larger surface areas degrade more rapidly than pellets, which have limited polymer-water interfaces and less space for microbial colonization (Volova et al., 2010).

The degradation of 3-PHB/3-PHV copolymers was faster than that of 3-PHB, with degradation rates increasing as the percentage of hydroxyvalerate in the copolymer rose. For example, in seawater, 58% of 3-PHB and 54% of 3-PHB/3-PHV films degraded after 160 days (Volova et al., 2010). It is worth noting that most P(3HB)- or P(3 HV)-degrading bacteria cannot degrade PHAs with six or more carbon atoms, suggesting that larger PHA monomers require specialized enzymes or microbes for degradation (Schirmer et al., 1993).

Additives, such as plasticizers used in polymer production to enhance physical properties like strength and flexibility, also affect degradation rates. While plasticizers improve PHB’s durability, they may alter its susceptibility to microbial activity. For instance, PHB films containing 10 and 20% tributyl citrate exhibited enhanced biodegradability. Tributyl citrate accelerated PHB degradation by 88% within the first day, although the final degradation levels after 3 days were comparable to non-plasticized films. By day three, all films achieved over 90% degradation. Despite slightly inhibiting the growth of *Microbulbifer* sp. SOL66, the degradation efficiency of plasticized PHB remained high (Cho et al., 2022). Adding plasticizers to PHB enhances its melt processability and optimizes its properties, which can potentially improve its biodegradability (Panaitescu et al., 2017). However, while some plasticizers are biodegradable, others are not. Thus, the choice of plasticizer significantly impacts the overall biodegradability of the polymer. Biodegradable plasticizers, such as certain citrate esters, can enhance the biodegradability of PHAs, whereas non-biodegradable plasticizers, like some phthalate-based compounds, may persist in the environment and potentially inhibit the biodegradation process (Meereboer et al., 2020). Therefore, while plasticizers can improve the biodegradability of PHAs by modifying their physical properties, the biodegradability of the plasticizers themselves is a crucial factor to consider in the design of environmentally friendly biopolymer systems. These findings highlight the critical interplay between environmental conditions, structural properties of PHAs, and microbial activity in determining biodegradation efficiency (Bonartseva et al., 2002).

Asummary of the factors influencing microbial PHA degradation is presented in Table 2.

**Table 2:** Factors influencing microbial PHA degradation.

Factor	Description	Impact on degradation
Oxygen Availability	Presence of oxygen (aerobic) or lack thereof (anaerobic).	Aerobic conditions result in carbon dioxide and water, while anaerobic conditions produce methane and carbon dioxide.
Temperature	Environmental temperature levels.	Higher temperatures enhance microbial activity and degradation rates; colder temperatures slow down the process.
Light	Exposure to light.	Increased light levels promote degradation by making polymers more accessible to microbial degradation.
Salinity	Salt concentration in the environment.	Higher salinity reduces PHA degradability.
pH	Acidity or alkalinity of the environment.	pH variations can influence microbial enzyme activity and thus affect degradation rates.
Nitrate Concentration	Availability of nitrate in the environment.	Nitrate enhances degradation by stimulating denitrifying microbes, especially under low-temperature conditions.
Polymer Properties	Shape, size, molecular weight, functional groups, crystallinity, and hydrophobicity/hydrophilicity.	Smaller size, lower molecular weight, and higher surface area (e.g., films vs. pellets) improve degradation rates.
Additives	Presence of plasticizers or other additives.	Plasticizers can improve physical properties but may also impact microbial activity; some accelerate degradation while others may inhibit it.
Polymer Type	Composition of the polymer (e.g., PHB, PHBV, PHBH).	Copolymers like 3-PHB/3-PHV degrade faster than homopolymers like 3-PHB; larger PHA monomers require specialized enzymes.
Carbon Source Availability	Presence of additional carbon sources such as lactose, glucose, etc.	Additional carbon sources can enhance microbial degradation, with lactose significantly boosting degradation efficiency.
Seasonal Variations	Changes in environmental conditions across different seasons.	Degradation rates are higher in summer due to warmer temperatures and increased microbial activity.

## Comprehensive overview of PHA depolymerases

PHA depolymerases (EC 3.1.1.75, EC 3.1.1.76) are enzymes that catalyze the degradation of PHAs, breaking them into smaller molecules such as oligomers, dimers, and monomers. These hydroxyalkanoic acid monomers serve as carbon and energy sources for microorganisms, playing an essential role in microbial life cycles and PHA recycling. PHA depolymerases also hold great promise for biotechnological applications, particularly in the sustainable management of bioplastics.

### Classification of PHA depolymerases

PHA depolymerases are categorized based on their localization and substrate specificity.

#### Localization

- *Extracellular (e-PHA depolymerases)*: Act on extracellular paracrystalline PHA granules.
- *Intracellular (i-PHA depolymerases)*: Degrade “native” PHAs stored as intracellular granules.
- *Periplasmic depolymerases*: Found in the periplasmic space of certain bacteria, playing roles in microbial metabolism and predation.

#### Substrate specificity

- *SCL-PHB Depolymerases*: Target short-chain-length PHAs such as poly (3-hydroxybutyrate) (PHB), producing 3-hydroxybutyrate monomers. Examples include depolymerases from *Rhodospirillum rubrum, Alcaligenes faecalis*, *Pseudomonas* spp., and *Bacillus* spp.
- *MCL-PHA Depolymerases*: Degrade medium-chain-length PHAs such as poly (3-hydroxyhexanoate) and poly (3-hydroxyoctanoate), with microbial producers like *Pseudomonas* spp.

*Intracellular PHA (i-PHA) depolymerases* degrade native PHAs stored as amorphous granules. Once the protective protein layer surrounding these granules is, PHAs denature into a paracrystalline form, becoming accessible to extracellular PHA depolymerases. Notably, *Pseudomonas lemoignei* produces an extracellular PHB depolymerase (PhaZ7), that uniquely degrades native PHB granules despite lacking significant similarity to conventional hydrolases (Braaz et al., 2003).

*Periplasmic PHA depolymerases*, though less studied, have been identified in *Rhodospirillum rubrum* and *Bdellovibrio bacteriovorus*. In *B. bacteriovorus*, these enzymes contribute to its predatory lifestyle by degrading host PHAs during its developmental cycle (Zampolli et al., 2024; Bonartseva et al., 2002; Handrick et al., 2004). In *R. rubrum*, the physiological role of this enzyme remains unclear, as its PHB granules are stored intracellularly rather than in the periplasm.

### Extracellular PHA depolymerase superfamilies

e-PHA depolymerases are further classified into two major superfamilies based on substrate specificity:

1. e-PHA_SCL_ depolymerases (EC3.1.1.75): Degrade short-chain-length PHAs (3 to 5 carbon atoms) including PHB, poly (3-hydroxyvalerate) (PHV), poly (3-hydroxybutyrate-co-3-hydroxyvalerate) (PHBV), poly (4-hydroxybutyrate) (P4HB), poly (3-hydroxybutyrate-co-4-hydroxybutyrate) (P(3HB-co-4HB)), poly(3-hydroxybutyrate-co-3-hydroxyvalerate-co-3-hydroxyhexanoate) (PHBVH), poly (3-hydroxybutyrate-co-3-hydroxy-methylpropionate) (P(3HB-co-3MP)), etc.
2. e-PHA_MCL_ depolymerases (EC3.1.1.76): Degrade medium-chain-length PHAs (6 to 14 carbon atoms) such as poly(3-hydroxyoctanoate) (P(3HO)), polyhydroxyoctanoate-co-hexanoate (P(HO-co-HH)) poly(hydroxyoctanoate-co-hydroxyhexanoate) (P(HO-co-HX)), poly(3-hydroxy-5-phenylvalerate) (PHPV), etc.

According to the PHA Depolymerase Engineering Database (Knoll et al., 2009), e-PHA depolymerases can be further categorized based on their catalytic domain:

- e-PHA_SCL_ depolymerases (Type 1 catalytic domain): Characterized by an N-terminal oxyanion hole.
- e-PHA_SCL_ depolymerases (Type 2 catalytic domain): Characterized by a C-terminal oxyanion hole.
- e-PHA_MCL_ depolymerases: Specific for medium-chain-length PHAs.
- e-PHA_SCL_ depolymerase: Only active against native PHA granules, with *Paucimonas lemoignei* as a notable representative (Handrick et al., 2001).

**Figure 1:**
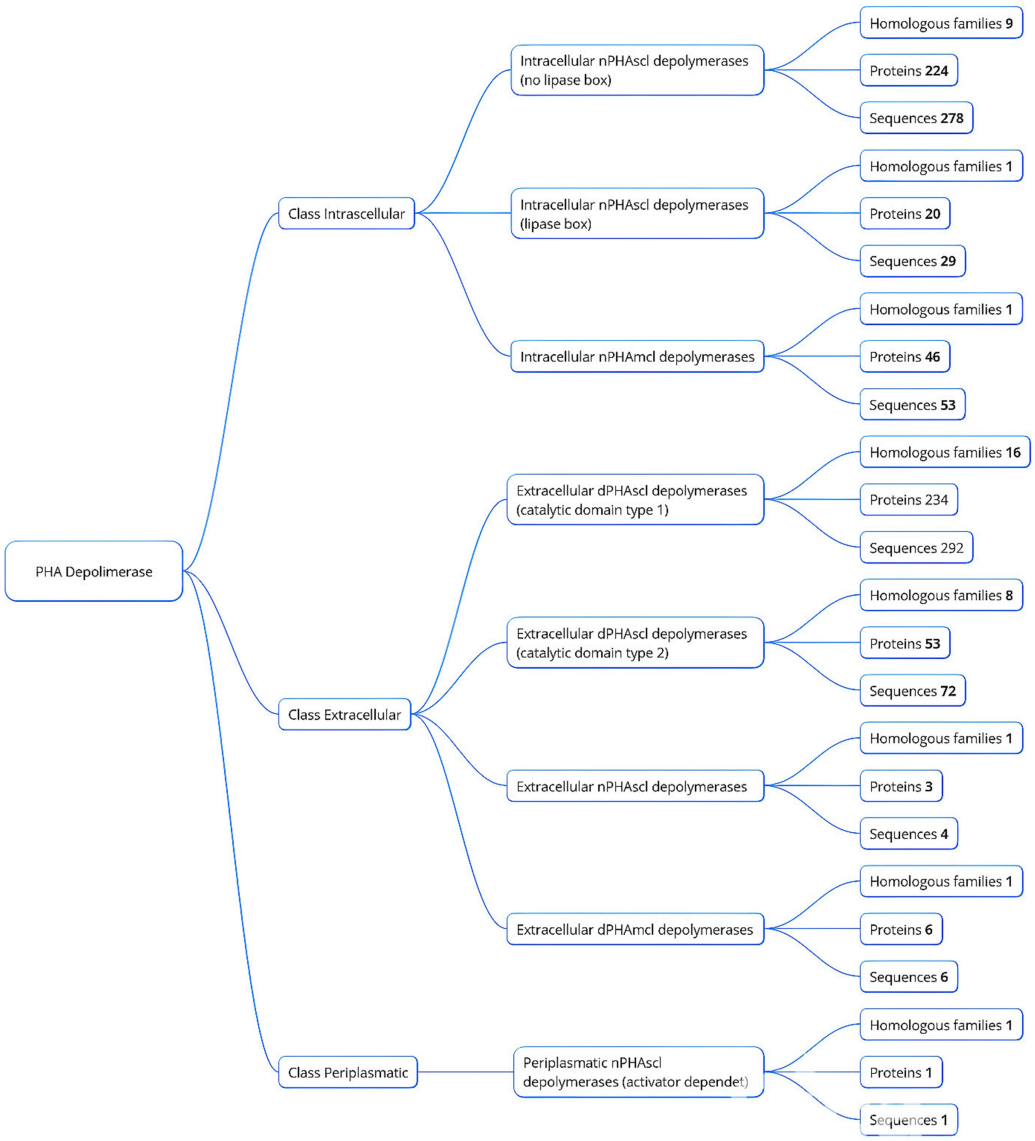
Classification of PHA depolymerases based on localization and homologous families.

Aschematic representation of this classification is shown in Figure 1, providing insights into the diversity and functionality of PHA depolymerases within microbial systems.

While sequence based identification of PHA depolymerases is widespread, only a fraction of these enzymes have been purified and functionally validated. Compared to extracellular PHB (e-PHB) depolymerases, the biochemistry of intracellular PHB (i-PHB) depolymerases remains largely unexplored. Further research is needed to elucidate the structural determinants of substrate specificity and the catalytic mechanisms of these enzymes.

Efforts to optimize the use of PHA depolymerases for applications such as bioplastic recycling, waste management, and the production of PHA monomers are still in the early stages. From a biotechnological standpoint, extracellular PHA depolymerases hold significant promise due to their ability to hydrolyze paracrystalline PHA granules into monomers and oligomers. These degradation products can be metabolized by other microorganisms, making e-PHA depolymerases especially valuable for:

- Bioplastic recycling: enzymatic breakdown of PHA-based polymers for reuse.
- Waste management: accelerating the degradation of biodegradable plastics.
- PHA monomer recovery: facilitating sustainable bioplastic production.

The potential of e-PHA depolymerases to advance the circular bioeconomy is substantial. Future research should focus on comparative studies of e-PHA_SCL_ and e-PHA_MCL_ depolymerases to optimize their industrial applications and improve our understanding of their ecological roles.

## Advances in the characterization and functional analysis of e-PHA_SCL_ depolymerases

To date, various e-PHA depolymerases exhibiting activity toward SCL-PHA have been isolated and characterized from diverse bacterial origins. These include enzymes from *Acidovorax* (Vigneswari et al., 2015; Kobayashi et al., 1999; Wang et al., 2012), *Aeromonas* (Amir et al., 2024), *Agrobacterium* (Guo et al., 2016), *Alcaligenes* (Nojiri and Saito, 1997; Saito et al., 1989; Kita et al., 1997), *Anoxybacillus* (Colak et al., 2005), *Arthrobacter* (Asano and Watanabe, 2001), *Aureobacterium* (Sadocco et al., 1997), *Bacillus* (Ma et al., 2011; Takaku et al., 2006), *Burkholderia* (Azura Azami et al., 2019), *Caldimonas* (Lee et al., 2018), *Comamonas* (Kasuya et al., 1997; Shinomiya et al., 2006; Jendrossek et al., 1995), *Leptothrix* (Takeda et al., 2000; Takeda et al., 1998), *Lihuaxuella* (Thomas et al., 2022), *Marinobacter* (Kasuya et al., 2003), *Microbacterium* (Sadocco et al., 1997; Sayyed et al., 2019), *Paucimonas* (Braaz et al., 2003; Jendrossek et al., 1993; Briese et al., 1994; Jendrossek et al., 1995; Schöber et al., 2000), *Pseudomonas* (Di et al., 2019; Li et al., 2016; Ohura et al., 1999; Uefuji et al., 1997; Mao et al., 2013), *Schlegelella* (Elbanna et al., 2004), *Streptomyces* (Klingbeil et al., 1996; García-Hidalgo et al., 2012; Calabia and Tokiwa, 2006; Allen et al., 2011; Shah et al., 2007; Blevins et al., 2018), *Thermus* (Papaneophytou et al., 2009) (Table 2).

Characterized e-PHA_SCL_ depolymerases exhibit molecular weights ranging from 35 to 63 kDa (Amir et al., 2024; Ma et al., 2011), and display optimal activity across broad pH (Knoll et al., 2009; Bhatt et al., 2011; Lalonde et al., 2025; Zandieh et al., 2024; Kunioka and Doi, 1990; Hablot et al., 2008; Lin et al., 2020; Amir et al., 2024; Allen et al., 2011) and temperature ranges (30–80°C) (Sayyed et al., 2019; Elbanna et al., 2004; Allen et al., 2011). Among these, the most thermostable enzyme is derived from *Schlegelella thermodepolymerans*, exhibiting activity up to 90°C, with an optimal range of 75–90°C and stability after 24 h at 70°C (Elbanna et al., 2004). Another notable example is the enzyme from *Streptomyces* sp. IN1, which remains stable at 80°C for over 15 min and maintains activity at pH 12.0, highlighting its dual extremophilicity and industrial relevance (Allen et al., 2011). While certain enzymes, such as those from *Schlegelella thermodepolymerans* and *Streptomyces* sp. IN1, demonstrate exceptional stability, the structural basis for these properties remains unclear. Understanding these features could enhance the engineering of robust enzymes for industrial applications.

e-PHA_SCL_ depolymerases are commonly inhibited by reducing agents like dithiothreitol (DTT) and *β*-mercaptoethanol (ME), the importance of disulfide bonds for enzymatic activity. They are also sensitive to serine hydrolase inhibitors (e.g., PMSF, DFP) and chelating agents (e.g., EDTA). Most mature enzymes are inhibited by non-ionic (Tween 20, Tween 80, Triton X-100) and anionic (SDS) detergents. Metal ions also modulate activity; inhibitory effects are observed with Mn^2+^, Fe^2+^, and Ni^2+^, while activating effects are seen with Ca^2+^, Mg^2+^, Na^+^, and K^+^, likely due to structural stabilization or cofactor roles (Table 3).

**Table 3:** Characteristics of e-PHA_SCL_ depolymerases from various microorganisms.

Strains	MW, kDa	pH, temp. Optimum	Substrats	Hydrolitic products	Activation	Inhibition	References
*Acidovorax* sp. TP4	50	8.5, 40–45	PHB, PPa ^a^	-	-	Triton X100, DFP, DTT	Kobayashi et al. (1999)
*Acidovorax* sp. HB01	43	7.0, 50	PHB, PHBV, P(3HB-co-4HB), PCL	-	Na^+^, K^+^, Ca^2+^	Zn^2+^,Mg^2+^,Mn^2+^, EDTA, ME	Wang et al. (2012)
*Aeromonas caviae* Kuk1	35	8.0, 35	PHB	-	-	EDTA, SDS, Tween-20 Triton X 100	Amir et al. (2024)
*Agrobacterium* sp. DSGZ	34	7.0, 50	PHB, PHBV, P(3HB-co-4HB), PCL^b^	DP1, DP2	-	Mn^2+^, Co^2+^, EDTA, TritonX-100, Tween80	Guo et al. (2016)
*Alcaligenes faecalis* T1	48–50		PHB	-	DTNB	DTT	Saito et al. (1989)
*Anoxybacillus gonensis* G2		7.5, 60	PHB	-	Mg^2+^, Na^+^, K^+^, Ca^2+^	KCN^c^, NaN_3_^d^, EDTA	Colak et al. (2005)
*Arthrobacter* sp. W6	47	8.5, 50	PHB	-	-	PMSF^e^, Hg^2+^, Ag^+^, Pb^2+^	Asano and Watanabe (2001)
*Aureobacterium saperdae*	42.7	8.0 45	PHB	DP1, DP2	-	DFP, DAN, DTT, PHMB^f^, NBS	Sadocco et al. (1997)
*Bacillus* sp. NRRL B-14911	63	9.0, 70	PHB	DP1	-	PMSF, DTT	Ma et al. (2011)
*Bacillus megaterium* N-18-259	62.3	9.0, 65	PHB PNPacetate, PNP-butyrate,	-	Mg^2+^, Ca^2+^	PMSF, DTT, Tween-20, Triton X-100, SDS	Takaku et al. (2006)
*Burkholderia cepacia* DP1	53.3	6.0, 45	PHB and P(3HB-co-3 HV)	-	PMSF	Tween 20, Tween 80	Azura Azami et al. (2019)
*Caldimonas manganoxidans*	42	-	PHB, PCL, PLA, PBSA	-	-	-	Lee et al. (2018)
*Comamonas acidovorans* YM1609	45	9.0, 37	-	DP1, DP2	-	DFP, PMSF, DTT, Tween 20	Kasuya et al. (1997)
*Leptothrix* sp. HS	43–46	8.0, 70	PHB	DP1	-	DTT	Takeda et al. (1998)
*Lihuaxuella thermophila*	**-**	9.0, 70	PHB, PHBH, PHBVH, PLA, PCL	DP1	K^+^, Ca^2+^	-	Thomas et al. (2022)
*Microbacterium paraoxydans* RZS6	40	7.0, 30	PHB	-	Ca^2+^ Mg^2+^	Fe^2+^, ME	Sayyed et al. (2019)
*Pseudomonas* sp. DSDY0501	57.9	9.0, 60	PHB	DP1	H_2_O_2_, ME	SDS, EDTA	Di et al. (2019)
*Pseudomonas stutzeri* YM1006,	60	7.0, 50	PHB	DP1, DP2	-		Uefuji et al. (1997)
*Pseudomonas mendocina* DS04-T	PHAase I 59.4	8.5, 50	PHB,PHBV,andP(3HB-co-4HB), PCL	-	-	Fe^2+^, ME	Mao et al. (2013)
	PHAase II	8.0, 50	PHB,PHBV,andP(3HB-co-4HB), PLA	-	Na^+^, K^+^	Fe^2+^, EDTA, ME	
*Schlegelella thermodepolymerans*	40.0	8.2, 80	PHB, P(3HB-co-3MP)	-	-	DFP, DTT, EDTA, NaN_3_, ME	Elbanna et al. (2004)
*Streptomyces exfoliatus* K10	49	8.5–9, 40	PHB	DP1	Mg^2+^, Ca^2+^	DTT, EDTA 10, Tween20, SDS	Klingbeil et al. (1996), García-Hidalgo et al. (2012)
*Streptomyces* sp. *MG*	41–43	8.5, 60	PHB PHBV PPL, PEA^g^ PES^h^	-	Cu^2+^, Co^2+^ Zn^2+^	Mn^2+^, Fe^2+^, Ni^2+^, and Ag^+^, PMSF	Calabia and Tokiwa (2006)
*Streptomyces* sp. IN1	62	12, 80	PHB, PHBV	DP1	NaN_3_, Triton X-100	PMSF, DTT, Tween 80	Allen et al. (2011)
*Streptomyces* sp. SFB5A	47	7.0–8.5	PHB, PHV, PHBV	DP1, DP2, DP3	Ca^+2^, Mg^+2^	EDTA, DTT	Blevins et al. (2018)
*Thermus thermophilus* HB8	42	8.0, 70	PHB	DP1	Ca^2+^, Mg^2+^	ΕDTA, NaN_3_, PMSF, Tween20, Tween 80, ME	Papaneophytou et al. (2009)

a Polypropiolactone.

b Polycaprolactone.

c Polypropiolactone potassium cyanide.

d Sodium azide.

e Phenylmethylsulfonyl fluoride.

f p-hydroxymercuricbenzoate.

g Poly(ethylene adipate).

h Poly(ethylene succinate).

**Figure 2:**
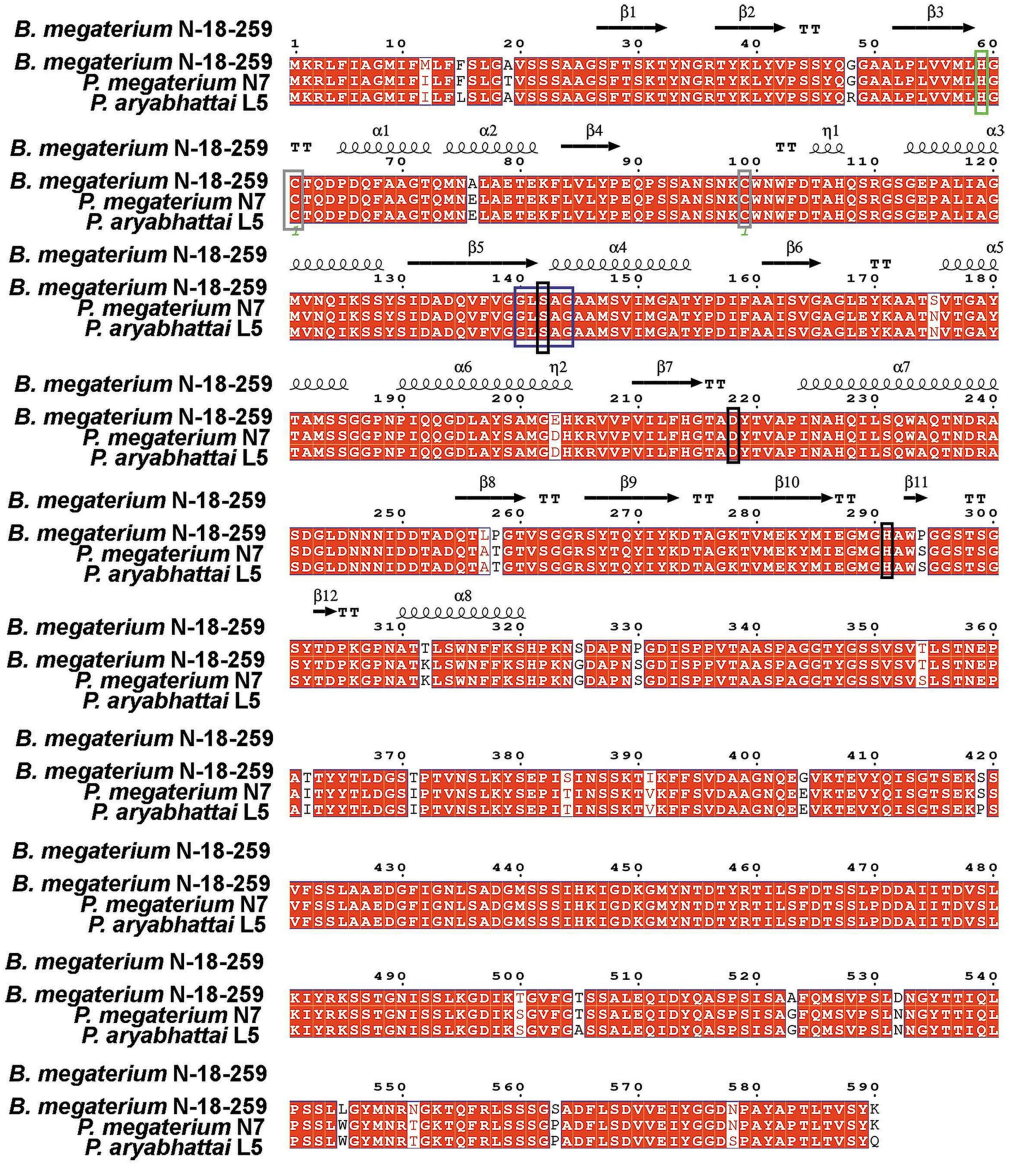
Comparative alignment of e-PHA_SCL_ depolymerase genes from *B. megaterium* N-18-25-9, *P. megaterium* N7, and *P. aryabhattai* L5, highlighting conserved catalytic triad, oxyanion hole, and structural motifs. The catalytic triad Ser146, Asp222, and His295 are marked in black, while the putative oxyanion hole residue His63 is highlighted in green. Two conserved cysteine residues are shown in brown, and the lipase box is marked in blue.

In the *Bacillus* genus, e-PHA_SCL_ depolymerases have been comprehensively studied only in *Bacillus* sp. strain B-14911 and *Bacillus megaterium* N-18-25-9 (Ma et al., 2011; Takaku et al., 2006). Our studies identified most strains as *Priestia megaterium* or *Priestia aryabhattai* (formerly *B. megaterium*). Whole-genome sequencing revealed that e-PHA_SCL_ depolymerase genes from *P. megaterium* N7 and *P. aryabhattai* L7 share 96% identity with *B. megaterium* N-18-25-9, with 22 amino acid differences suggesting variations in biochemical properties (Figure 2).

Our findings show that these enzymes degrade not only PHB but also copolymers like PHBV and PHBH. Interestingly, other strains exhibit different substrate specificities, underscoring the biochemical diversity of e-PHA_SCL_ depolymerases in *Bacillus*.

### Mechanism of depolymerization

PHA depolymerization occurs in two steps: (Covello et al., 2024) adsorption of the binding domain onto the PHA surface, and (Yadav and Nikalje, 2024) hydrolysis of polyester chains by the catalytic domain (Numata et al., 2009). Enzyme-substrate interaction does not necessarily guarantee hydrolysis. For instance, PHA depolymerase from *Alcaligenes faecalis* adsorbs to five different substrates—PHB, PHP, P(4HB), P(2HP), and P(6Hx), but hydrolysis occurs only with PHB, PHP, and P(4HB). This suggests that the substrate-binding domain functions independently of the catalytic domain and exhibits broader interactions (Kasuya et al., 1996).

Polymer chain scission begins with endo-scission (random cleavage along the chain) followed by exo-scission (cleavage from the chain ends).

While all characterized e-PHA_SCL_ depolymerases degrade PHB some also degrade PHV and other copolymers, though they generally prefer PHB. Notably, PhaZ6 e-PHA_SCL_ depolymerase from *Pseudomonas lemoignei* displays higher activity toward PHV than PHB (Schöber et al., 2000). Understanding such substrate preferences could inform enzyme engineering for expanded specificity. Most of the characterized enzymes hydrolyze SCL-PHA down into monomers (Thomas et al., 2022; Di et al., 2019; Ma et al., 2011; Takeda et al., 1998; Klingbeil et al., 1996; García-Hidalgo et al., 2012; Allen et al., 2011), while others generate monomers, dimers and trimers (Guo et al., 2016; Sadocco et al., 1997; Kasuya et al., 1997; Uefuji et al., 1997; Blevins et al., 2018).

### Catalytic and structural features

e-PHA_SCL_ depolymerases belong to the serine hydrolase family, characterized by the lipase-box motif Gly-X1-Ser-X2-Gly, where X1 is leucine/isoleucine and X2 is alanine/serine (Lee et al., 2018). The catalytic triad (Ser, Asp., His) and oxyanion hole (often cysteine-based) are critical for activity. Variations in these motifs suggest potential functional diversity. Many e-PHA_SCL_ depolymerases share similarities with lipases and esterases. However, Thomas et al. recently reported an e-PHA_SCL_ depolymerase from *Lihuaxuella thermophila* that resembles proteases and esterases but not lipases. This enzyme’s catalytic triad consists of Ser121, His270, and Asp197, while its oxyanion hole is formed by Cys40 (Thomas et al., 2022). The functional significance of motif variations remains poorly understood.

**Figure 3:**
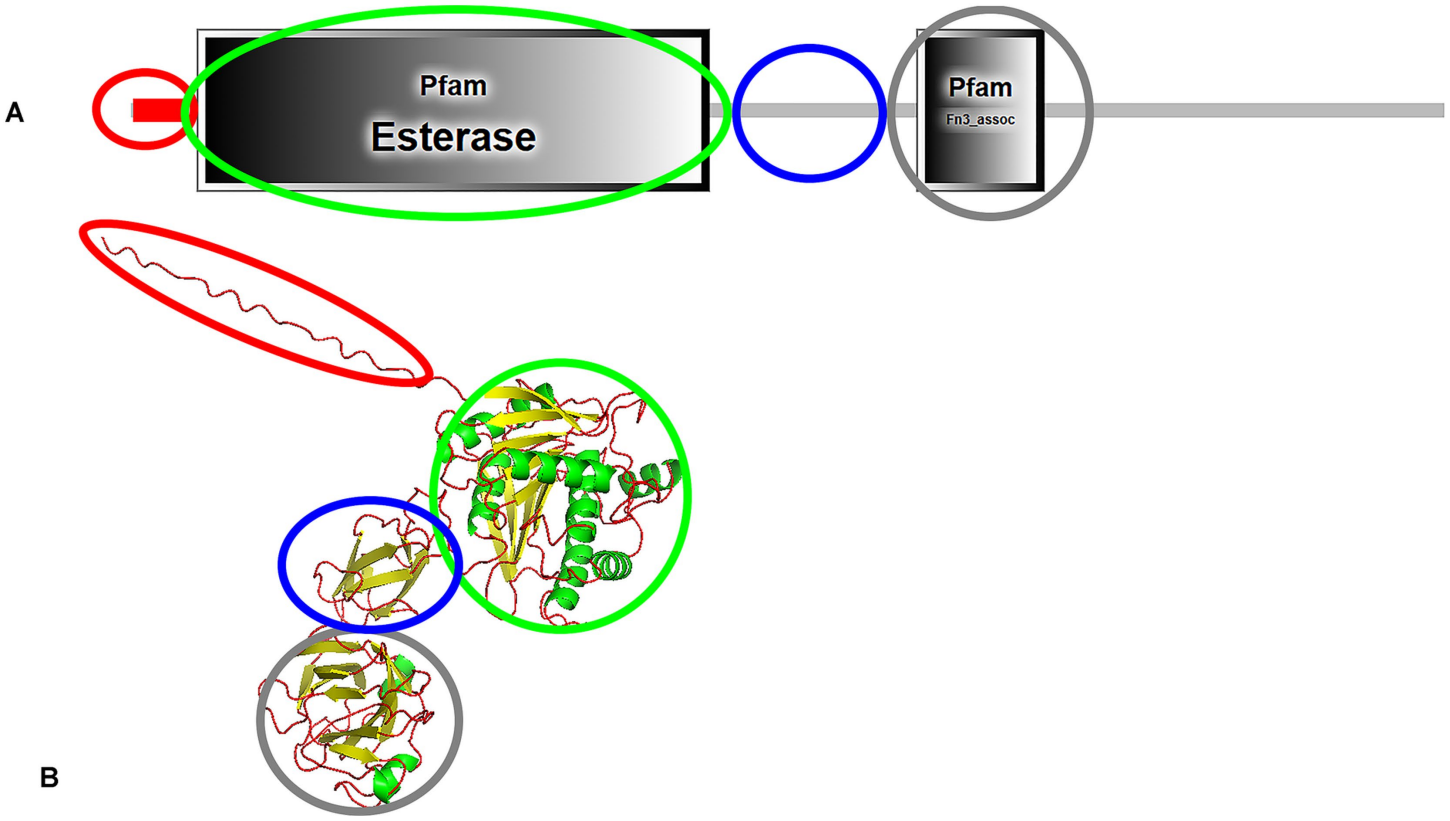
Domain architecture **(A)** and predicted structure **(B)** of e-PHA_SCL_ depolymerase from *Bacillus megaterium* N-18-25-9, highlighting functional domains. The signal peptide is marked in red, the catalytic domain in green, the linking domain in blue, and the C-terminal substrate-binding domain in grey. The structure was analyzed using SMART protein analysis and predicted by AlphaFold3.

Most e-PHA_SCL_ depolymerases have four domains: signal peptide, catalytic domain, linker domain, and C-terminal substrate-binding domain (Ma et al., 2011; Takaku et al., 2006). The domain architecture of the e-PHA_SCL_ depolymerase from *Bacillus megaterium* N-18-25-9, identified through SMART protein analysis (Letunic et al., 2009) and modeled by AlphaFold3 (Abramson et al., 2024), is presented in Figure 3. Interestingly, the SMART database did not predict a distinct linking domain, a long linking region is observed, suggesting linker function.

Two types of catalytic domains (CD) exist: Type I, where the lipase-box is located in the middle of the sequence, and Type II, where it is near the N-terminus. Three types of linker domains—Fibronectin Type III (Fn3), Threonine-Rich (Thr-rich), and Cadherin-Like (Cad)—and two types of substrate-binding domains (SBD1 and SBD2) have been characterized (Jendrossek and Handrick, 2002). *Bacillus* derived e-PHA_SCL_ depolymerases possess a Type I catalytic domain (Ma et al., 2011; Takaku et al., 2006), while *Comamonas acidovorans* YM1609 and *Leptothrix* sp. strain HS feature Type II domains (Kasuya et al., 1997; Takeda et al., 1998). Despite structural variations in the catalytic domain—specifically, the position of the oxyanion hole—no significant differences in substrate specificity have been observed. This statement highlights an intriguing aspect of PHA depolymerase functionality. Regarding linker domains: *Streptomyces* sp. SFB5A, *Comamonas acidovorans* YM1609, and *Leptothrix* sp. strain HS contain an Fn3-type linking domain (Kasuya et al., 1997; Takeda et al., 1998; Blevins et al., 2018), *Marinobacter* sp. NK-1 has a Cad-type linking domain (Kasuya et al., 2003) and *P. lemoignei* features a Thr-rich linking domain (Briese et al., 1994). Notably, in *Pseudomonas stutzeri*, the linking domain has not been identified (Ohura et al., 1999). While different types of linking (e.g., Fn3, Cad, and Thr-rich) and substrate-binding domains (SBD1 and SBD2) have been characterized, their roles in substrate recognition, binding efficiency, and catalytic enhancement remain unclear. In some e-PHA_SCL_ depolymerases, such as in *Pseudomonas stutzeri*, the linking domain is absent, raising questions about alterantive structural mechanisms compensating for its absence. Limited availability of three-dimensional structures in the Protein Data Bank constrains our understanding of these enzymes.

The 3D structure of the PHB depolymerase PhaZ7 from *Paucimonas lemoignei*, which is active against amorphous PHB, has been extensively analyzed. This study revealed significant differences in substrate-binding strategies, active site accessibility, and conformational flexibility (Papageorgiou et al., 2008). Critical amino acid residues essential for substrate binding and enzymatic activity were identified, emphasizing a unique substrate-binding domain distinct from the catalytic core. Through mutagenesis, binding assays, and high-resolution structural analysis, researchers demonstrated how specific mutations influence PHB degradation (Jendrossek et al., 2013). More recently, the structure of a PHB depolymerase from the thermophilic *Lihuaxuella thermophila* provided further insights into broad substrate specificity, including activity against PLA and PCL. This enzyme’s shallow active site cleftfacilitates diverse substrates access, contrasting with *P. lemoignei* PhaZ7, where a mobile loop occludes the active site, reducing versatility (Thomas et al., 2022).

## Advances in the characterization and functional analysis of e-PHA_MCL_ depolymerases

Microorganisms capable to degrade MCL-PHAs are less commonly found in the environment, and only a few e-PHA_MCL_ depolymerases have been characterized to date. Most of the known e-PHA_MCL_ depolymerases have been identified in species of *Pseudomonas* (Schirmer et al., 1993; Elbanna et al., 2004; Schirmer and Jendrossek, 1994; Schirmer et al., 1995; Kim et al., 2002) and *Streptomyces* (Martínez et al., 2015; Kim, 2003; Gangoiti et al., 2012; Santos et al., 2013). However, e-PHA_MCL_ depolymerases have also been characterized in other genera, such as *Bdellovibrio* (Martínez et al., 2012), *Thermus* (Papaneophytou et al., 2011), *Xanthomonas* (Kim et al., 2000). The first e-PHA_MCL_ depolymerase studied in detail at the molecular level was from *Pseudomonas fluorescens* GK13 (Schirmer et al., 1993; Schirmer and Jendrossek, 1994; Schirmer et al., 1995). Detailed results of charachterised e-PHA_MCL_ are presented in Table 4.

**Table 4:** Characteristics of e-PHA_MCL_ depolymerases from various microorganisms.

Strain	MW, kDa	pH, temp. Optimum	Substrats	Esterase activity	Hydrolitic products	Activation	Inhibition	References
*Bdellovibrio bacteriovorus* HD100	29.8	10.0, 37	P(HO-co-HX), PHACOS	−	DP2	−	PMSF, DTT, Tween 80, Triton X-100, SDS	Martínez et al. (2012)
*Pseudomonas fluorescens* GK13	25–26	8.0, 30–32	P(3HO) P(3HD-co-3PO)	+	DP2	−	−	Schirmer et al. (1993), Schirmer and Jendrossek (1994), Schirmer et al. (1995)
*Pseudomonas indica* K2	28	8.5, 35	P (3HO)	−		−	PMSF DFP, NaN3, EDTA	Elbanna et al. (2004)
*Pseudomonas alcaligenes* M4-7	28.0	9.0, 35	P(3HO)	−		−	DFP, PMSF, PHMB EDTA	Kim et al. (2005)
*Pseudomonas alcaligenes* LB19	27.6	9.0, 45	aliphatic and aromatic MCL-PHAs	+	DP1	−	EDTA, PMSF, NaN_3_ Tween 80, Triton X-100	Kim et al. (2002)
*Streptomyces* sp. KJ-72	27.1	8.7, 50	C6-C11 MCL-PHAs, PCL,	+	DP2	−	Tween 80, Triton-X 100, DFP	Kim (2003)
*Streptomyces exfoliatus* K10 DSMZ 41693	27.6	10.0, 30	P(HO-co-HH)		DP1	SDS, Tween20, TritonX-100	Mg^2+^ Ca^2+^, EDTA	Martínez et al. (2015)
*Streptomyces roseolus* SL3	28	9.5,	P(3HO)	+	DP1	−	−	Gangoiti et al. (2012)
*Streptomyces venezuelae* SO1	27	9.0, 50	P(3HO)	+	DP1	TritonX-100, Tween80, SDS, Propanol	DTT, EDTA	Santos et al. (2013)
*Thermus thermophilus* HB8	24–28	8.5, 70	P (3HO) and P(3HD-co-3HO)	+	−	Aceton, methanol	PMSF, NaN_3_, EDTA, Triton X-100, Tween 80, Tween 20, ME	Papaneophytou et al. (2011)
*Xanthomonas* sp. JS02	41.7	8.0, 60	PHPV	−	−	−	−	Kim et al. (2000)

The molecular weight of e-PHA_MCL_ depolymerases generally ranges between 25 and 30 kDa (Schirmer et al., 1993; Schirmer and Jendrossek, 1994; Schirmer et al., 1995; Gangoiti et al., 2012; Martínez et al., 2012). However, to the best of our knowledge, the largest reported e-PHA_MCL_ depolymerase derived from *Xanthomonas* sp. JS02 has a molecular weight of 41.7 kDa (Kim et al., 2000).

e-PHA_MCL_ depolymerases exhibit pH optima between 8.0 and 10.0 and temperature optima ranging from 30°C to 70°C. The most thermostable e-PHA_MCL_ depolymerase identified to date is from *Thermus thermophilus* HB8, with an optimum temperature of 70°C. In contrast, the e-PHA_MCL_ depolymerases of *P. fluorescens* GK13 (Schirmer et al., 1993; Schirmer and Jendrossek, 1994; Schirmer et al., 1995) and *Pseudomonas indica* K2 (Elbanna et al., 2004) are most active at 30–35°C. Interestingly, dithiothreitol (DTT), which reduces disulfide bonds, does not affect the activity of e-PHA_MCL_ depolymerases derived from *Pseudomonas* (Schirmer et al., 1993; Schirmer and Jendrossek, 1994; Schirmer et al., 1995; Kim et al., 2002). However, the e-PHA_MCL_ depolymerases of *Streptomyces venezuelae* SO1 (Santos et al., 2013) and *Bdellovibrio bacteriovorus* HD100 (Martínez et al., 2012) are inhibited by DTT. This suggests that essential disulfide bonds are required for the enzymatic activity of these depolymerases, unlike those from *Pseudomonas*, which do not rely on such bonds for functionality. In some cases, EDTA exhibits an inactivating effect (Martínez et al., 2015), while in other cases, it has no impact (Kim, 2003). This suggests that the e-PHA_MCL_ depolymerases are either not metalloenzymes or that the metal ions essential for activity are enclosed in a manner that prevents EDTA from chelating them. It is well established that e-PHA_MCL_ depolymerases are serine hydrolases, which in most cases are inactivated by PMSF. Interestingly, e-PHA_MCL_ depolymerases *Streptomyces* genera show minimal or no inhibition by PMSF compared with *Pseudomonas* (Martínez et al., 2015; Elbanna et al., 2004; Santos et al., 2013; Papaneophytou et al., 2011). This suggests that the structural architecture of these *Streptomyces* enzymes may differ significantly from that of *Pseudomonas* derived e-PHA_MCL_ depolymerases, explaining their resistance to this inhibitor. Typically, e-PHA_MCL_ depolymerases are inhibited by detergents. However, the activity of the enzymes from *Streptomyces exfoliatus* K10 and *S. venezuelae* SO1 demonstrated a notable enhancement in the presence of low concentrations of nonionic and anionic detergents, particularly with 0.01% SDS (Martínez et al., 2015; Santos et al., 2013). Most studied e-PHA_MCL_ depolymerases are not activated by metals such as Ca^2+^ and Mg^2+^, suggesting that these enzymes do not require metal ions as cofactors for their activity (Schirmer et al., 1993; Schirmer and Jendrossek, 1994; Schirmer et al., 1995; Kim et al., 2002; Martínez et al., 2012). Furthermore, the depolymerase activity of *S. exfoliatus* was notably inhibited by CaCl₂ and MgCl₂ (Martínez et al., 2015).

Regarding the substrate polymers, most of the characterized enzymes exhibit activity toward MCL-PHA, degrading them into monomers (DP1) (Martínez et al., 2015; Kim et al., 2002; Gangoiti et al., 2012; Santos et al., 2013), However, enzymes from *P. fluorescens* GK13 (Schirmer et al., 1993; Schirmer and Jendrossek, 1994; Schirmer et al., 1995), *Streptomyces* sp. KJ-72 (Kim, 2003) and *Bdellovibrio bacteriovorus* HD100 (Martínez et al., 2012) primarily hydrolyze P(3HO) into dimeric forms (DP2). The enzyme from *S. exfoliatus* K10 DSMZ 41693 has been reported to function as an endo-exo-hydrolase, capable of cleaving both large and small PHA molecules. Remarkably, it requires only 30 min to produce monomers, whereas other enzymes demand higher enzyme loading and longer reaction times to achieve similar results (Gangoiti et al., 2012; Santos et al., 2013).

Some e-PHA_MCL_ depolymerases also exhibit versatile, with applications extending to the hydrolysis of copolymers. For instance, the recombinant e-PHA_MCL_ depolymerase from *P. fluorescens* GK13 can successfully degrade copolymer consisting of PHB and polyhydroxyoctanoate (PHO) (Millan and Hanik, 2023). Additionally, the e-PHA_MCL_ depolymerase from *S. exfoliatus* K10 has been applied to hydrolyze functionalized polymers as PHACOS, yielding functional thioester-based monomers (Martínez et al., 2015).

The amino acid sequence of characterized e-PHA_MCL_ depolymerases varies between 277 and 282 residues. These enzymes contain high proportion of amino acids with aromatic and uncharged aliphatic side chains, contributing to their hydrophobic nature. The amino acid sequence similarity among characterized e-PHA_MCL_ depolymerases ranges from 69 to 98% (Kim et al., 2005). Sequences analysis revealed that they generally consist of a signal peptide, an N-terminal substrate-binding domain, and a C-terminal catalytic domain (Kim et al., 2002; Gangoiti et al., 2012; Papaneophytou et al., 2011). However, in the case of *P. fluorescens* GK13, only the signal peptide was identified, with substrate-binding domain likely located in N-terminal region (Schirmer and Jendrossek, 1994; Jendrossek and Handrick, 2002; Kim et al., 2005). Unlike e-PHA_SCL_ depolymerases, no linking domain has been identified between the substrate-binding region and the catalytic domain in e-PHA_MCL_ depolymerases. e-PHA_MCL_ depolymerases contain a conserved catalytic triad (Ser, Asp., His) in the active center (Martínez et al., 2015; Gangoiti et al., 2012). The catalytic domain of all e-PHA_MCL_ depolymerases contains a lipase box (Gly-X1-Ser-X2-Gly), where X1 is an Ile and X2 is a Ser (Martínez et al., 2012). However, variations have been observed: in*T. thermophilus*, X1 is Gly and X2 is Tyr (Papaneophytou et al., 2011), in *S. exfoliatus* and *S. roseolus*, X1 is His and X2 is Gln (Martínez et al., 2015). Additionally, all PHA depolymerases contain an oxyanion hole residue essential for stabilizing the transition state during hydrolysis. This residue varies across species, being His in *P. fluorescens* GK13 (Schirmer and Jendrossek, 1994), Ser in *Pseudomonas alcaligenes* M4-7 (Kim et al., 2005), Asn111 in *P. alcaligenes* LB19 (Kim et al., 2005), Asn10 in *T. thermophilus* (Papaneophytou et al., 2011), and Gln147 in *Streptomyces roseolus* SL3 (Gangoiti et al., 2012).

Further ecological studies on MCL-PHA degraders, along with advancements in the biochemistry and molecular biology of MCL-PHA depolymerases, are essential to expanding our understanding of MCL-PHA degradation and its potential applications.

The vast majority of PHA-degrading microorganisms are known to produce only one type of PHA depolymerase acting upon either SCL-PHAs or MCL-PHAs. Very few bacterial strains have been identified with both e-PHA_SCL_ depolymerase and e-PHA_MCL_ depolymerase activities. One such strain is *Pseudomonas indica* K2. Researchers demonstrated that this strain produces two distinct depolymerases: one specific for SCL-PHA and another for MCL-PHA. Additionally, they successfully purified and characterized the extracellular poly (3HO) depolymerase, which exhibited activity for MCL-PHA substrates but not for SCL-PHB, further confirming its role as an e-PHA_MCL_ depolymerase (Elbanna et al., 2004). Similar results have been found for *Xanthomonas* sp. JS02 (Kim et al., 2000), *S. exfoliatus* K10 (Klingbeil et al., 1996; García-Hidalgo et al., 2012). Another example is *Streptomyces* sp. KJ-72 strain which also produces e-PHA_MCL_ depolymerase. Interestingly it is expressed only if PHO was used as a carbon source in the cultivation media. It has also been reported that if PHB is used as a source of carbon PHB depolymerase is being produced (Kim, 2003). Additionally *T. thermophilus* produces an extra cellular e-PHA_MCL_ depolymerase. In addition, it has been previously demonstrated that *T. thermophilus* is able to produce e-PHA_SCL_ depolymerase suggesting that this microorganism secretes different PHA depolymerases depending on the growth conditions. The extracellular e-PHA_SCL_ depolymerase of *T. thermophilus* was found to be secreted constitutively in the presence of MCL-alkanoates but not in the presence of glucose or SCL-alkanoates, similarly to the extracellular PHO depolymerase of *P. fluorescens* GK13 (Schirmer et al., 1993). According to Schirmer et al. high expression of the e-PHA_MCL_ depolymerase depends on (i) the presence of 3HAs or similar compounds (as inducers) or (ii) carbon starvation. Thus, satisfactory amounts of PHA depolymerase can be produced using sodium octanoate as sole carbon source instead of PHO (Schirmer et al., 1993).

## Mechanisms of the enzymatic degradation of PHA in the environment

**Figure 4:**
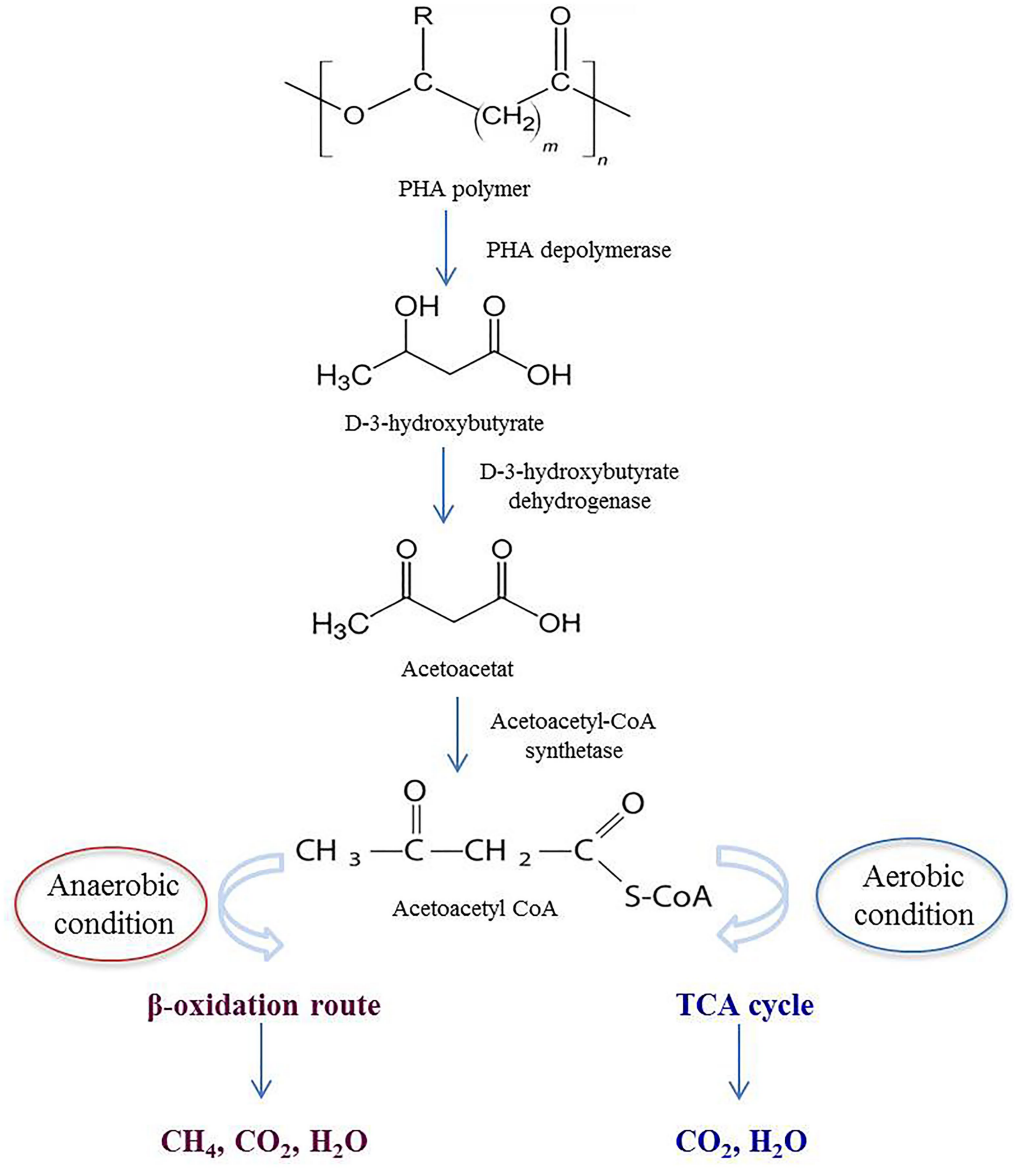
Schematic depiction of extracellular PHA degradation in both anaerobic and aerobic environments.

When PHA is disposed in the environment, it comes into contact with natural microbial communities. Specific microorganisms secrete PHA depolymerases, which catalyze the hydrolysis of ester bonds in the polymer, breaking it down into smaller units, primarily D-3-hydroxybutyrate and acetoacetate. These monomers or oligomers are taken up by the microorganisms and enter the microbial metabolic pathways, such as the *β*-oxidation pathway in anaerobic bacteria, or tricarboxylic acid cycle (TCA) cycle in aerobic bacteria. As a result, the monomers are further broken down into fundamental metabolites like acetyl-CoA. The final metabolic products include carbon dioxide (CO_2_), water (H_2_O), and microbial biomass under aerobic conditions. In anaerobic environments methane (CH_4_) may also be produced alongside CO_2_ (Figure 4) (Numata et al., 2009; Zhou et al., 2023).

Unlike conventional plastics, PHA degrades into environmentally benign by-products without leaving toxic residues. The by-products contribute to the natural carbon cycle, potentially enhancing soil fertility or water quality (Shah et al., 2008). While the effectiveness of depolymerases has been well demonstrated in controlled laboratory conditions, their performance in real-world environments, such as landfills and soil, is significantly influenced by various environmental factors often not considered in laboratory studies, though degradation rates are generally lower compared to controlled settings. Studies have shown that microbial activity is the primery driver of PHA degradation with specific bacteria such as *Pseudomonas* and *Acidovorax* playing key roles. Additionally, degradation rates vary depending on the monomer composition of the PHA. For instance, P(3HB-co-4HB) degrades more rapidly than P(3HB) and P(3HB-co-3 HV). It has been reported that certain copolymers exhibit degradation rates of 98.9 ± 1.8% in soil and 81.5 ± 2.9% in lake environments after five weeks (Sevakumaran et al., 2019).

Furthermore, PHBV has shown significant biodegradation under compost and soil conditions. In controlled compost, PHBV degraded by 90% within 200 days, whereas in soil, the degradation rate was only 32% over the same period (Muniyasamy et al., 2016). Additionally marine microorganisms have been reported to degrade PHAs by secreting depolymerases that break down the polymer (Hachisuka et al., 2023).

However, the lack of standardized testing methods for deep-sea conditions presents challenges in accurately assessing PHA biodegradability in such environments (Meereboer et al., 2020).

## Summary and future perspectives

For a successful bioindustrial process, enzymes must exhibit fast kinetics, long-term stability, and high activity within the specific environmental context. Special attention should be given to enzymes that are stable at high temperatures and resistant to various salts and detergents, as these properties are crucial for their functionality under harsh production conditions. Extremophilic microorganisms are valuable resources for identifying and engineering robust PHA depolymerases for industrial and environmental applications. Despite notable advancements in the purification and biochemical characterization of PHA depolymerases, structural studies remain limited. The lack of detailed 3D structural information hinders our understanding of critical mechanisms, such as enzymes-substrate recognition, binding and degradation, as well as how they maintain activity under extreme environmental conditions. By further advancing our understanding of microbial and enzymatic degradation processes, we can pave the way for a circular plastic bioeconomy, establishing PHAs as a viable and eco-friendly alternative to petroleum-based plastics.
